# A New Function of the Placenta, Lately Discovered by Prof. Claude Bernard

**Published:** 1859-07

**Authors:** 


### EDITORIAL DEPARTMENT.

### *A New Function of the Placenta, lately discovered by Prof. Claude Bernard.*

Science, every day, feels the efforts of this indefatigable physiologist; and we now have, as the fruit of his labors, a solution of the before inexplicable phenomenon of the presence of sugar in the amniotic fluid, during the first months of intra-uterine existence, before it appears in the tissue of the liver, and its absence after the liver has taken on that function which is now shown to be so important and universal. The placenta has now been demonstrated by Bernard to have a sugar-producing function added to that of aërating and nourishing the blood of the fœtus. The article in which this discovery is embodied, is to be found in the last number of the "*Journal de la Physiologie*," and is so interesting that we propose to make it the subject of a brief analysis.

Bernard regards the functions of the placenta, as quite various and complicated; the opinion that it was mainly the respiratory organ of the fœtus, being founded on the fact that the office of the lungs commences where that of the placenta is arrested; giving the appearance of a substitution one for the other. Without going into the various functions of the placenta, it is the object of this article to demonstrate anatomically and physiologically, that the placenta is destined to perform the glycogenic office of the liver in the first months of development; before that organ was acquired the development of its structure which will permit it to perform that function. The author has already shown that the sugar producing function of the liver does not commence until the development of the fœtus is somewhat advanced; and, on the other hand, that it is impossible that the glycogenic matter should come from the mother, in the mammalia; which fact is even more indubitable in birds, in which the young is separately developed. It was evident, therefore, that this function was divided among different organs of the body, or temporarily localized in some intra-uterine organ, which disappeared when the liver took on its functions.

The first experiments on this subject were made upon the placentæ of ruminant animals, which were the most easily obtained, and were entirely without success; it being impossible to find any part of them which contained glycogenic matter. The placentæ in the ruminants, it will be remembered, are multiple, with an intervening membrane. In spite of this want of success, Bernard had recourse to the placentæ of rabbits and Guinea-pigs. He then found a whitish substance composed of agglomerated epithelial and glandular cells; and established that these, like those of the liver, were filled with a glycogenic material. This mass of cells seemed to be situated between the maternal and fœtal portions of the placenta; and after having been developed, to become atrophied as the fœtus approached the period of birth. He thus ascertained that the placenta of the rabbit and Guinea pig, was composed of two portions having distinct functions. The experiments, however, which had been made upon such a number of the ruminant animals seemed to make it necessary to admit that they were exceptions to the physiological rule. The word exception, Mr. Bernard very shrewdly remarks, is generally used merely to cover over ignorance of the real condition of a phenomenon. Believing that there was only a variation in the disposition of the glycogenic portion of the placenta in the ruminants, and not a complete absence of it, Bernard renewed his experiments, this time with complete success. He established that in animals with a single placenta, the vascular and glandular portions of that organ are mingled; but in the ruminants, that these are situated in distinct membranes and can, therefore, be studied separately. Thanks to this anatomical arrangement, we can clearly prove that the vascular portion of the placenta persists and grows, up to the time of birth; while the glandular portion increases during the first period of gestation, attains its maximum about the third or fourth month of intra-uterine existence,* then gradually disappears in passing through the various forms of atrophy and degeneration, until, at the birth of the mammalia, there no longer exists a trace of this temporary hepatic portion of the placenta. But it must be added, that during the time which this hepatic portion of the placenta is growing, the liver possesses neither its structure nor its functions, and that it is precisely at the time that the liver is developed, and its cells, having acquired their definite form, commence to secrete glycogenic matter, that the hepatic organ of the amnios tends to disappear.

The hepatic patches of the amnios, in ruminant animals, appear from the beginning of embryonic life. They are developed little by little on the internal face of the amnios, covering the umbilical cord up to a point where a distinct line separates the skin from the amnios. Afterwards, these patches which, over the portion covering the cord, take the form of villo-

---

* These limits can only be given approximatively on account of the impossibility of ascertaining the exact age of the calves which are obtained at the slaughter-houses.

sities, extend over the other portions of the amnios, *pari passu* with the development of the blood-vessels which accompany them. They augment little by little in volume; formed in the first place of a transparent matter, they become later more opaque, especially near their borders, which are a little elevated, and sometimes resemble, in their aspect, patches of lichen. They are at other times, of various flattened or filiform shapes, and run into each other in such a manner as to become confluent. At their full development, the patches present a thickness which is sometimes as great as three or four millimetres (about one-eighth to one-sixth of an inch); those which are filiform present often a much greater length, and are sometimes united in the form of a mass at their extremities. At a later period, these hepatic patches of the amnios cease to develop themselves. In certain parts they become yellowish, of a fatty appearance; in other situations they fall and float in the amniotic liquid, and leave on the membrane a kind of cicatrix which afterwards completely disappears. The mode of degeneration and disappearance of the hepatic patches of the amnios appear to be quite varied. When they disappear by desquamation and complete resorption of the cicatrix, no trace of the patches can be found at the birth of the fœtus. When fatty degeneration affects the adherent patches, they are found at the birth of the fœtus transformed into fat, and sometimes considerably thickened. It sometimes may happen, in this case, that some of these fatty masses detach themselves from the amnios and float in the amniotic fluid.

One can establish with the greatest facility the presence of a glycogenic matter in the hepatic patches of the amnios at all periods of their development. From their first appearance, it is easy to recognize this matter under the microscope, with the aid of iodine. When the patches are completely developed, it is easy to extract the glycogenic matter in considerable quantity, and study its characters.

We have made an almost literal translation of the above portion relating to the position, development, and disappearance of these "hepatic patches of the amnios" in the ruminant animals. Here, as the author remarked, they can be studied by themselves, completely isolated from the other portions of the placenta. The process of separating the glycogenic matter is extremely simple, consisting merely of separating the patches from the membrane by the means of boiling water, and extracting the saccharine matter by ebullition, after having bruised the patches in a mortar, in the same manner as we proceed to extract the same substance from the tissue of the liver. When extracted, it offers precisely the same character, as the saccharine matter of the liver. It is dissolved in water, giving it a milky appearance, is precipitated by alchohol and by acetic acid.

Iodine gives it an intense wine-red color, which is destroyed by heat, and re-appears on cooling. It is also changed into dextrine and fermentable sugar under the influence of animal and vegetable ferments, and by boiling with strong acids.

The amniotic membrane in the calf, seems at first to be devoid of a well-marked epithelium; but when the patches appear, we can make out, by means of the microscope, on its internal face, and first in the part which covers the umbilical cord, spots formed by epithelial cells; afterwards, in the center of these spots, clusters of glandular cells are seen, small in number at first, sometimes only one or two, even, in a spot. These glandular cells may be distinguished from the others by their form and their reaction with iodine. If we add to one of these amniotic plates, placed in the field of the microscope, a little tincture of iodine slightly acidulated with acetic acid, we will see immediately the glycogenic cells take the wine-red color, while the epithelial cells remain colorless or become slightly yellow. Little by little the groups of glycogenic cells by their development augment and take the form of papillæ, particularly on that part of the membrane which covers the cord. Examined by the microscope, these papillæ are composed of glycogenic cells covered by an epithelium. The hepatic plates are composed of the same element.

Isolated cells may be obtained and examined by breaking up one of the patches. They contain a nucleus, sometimes with a nucleolus, and granular matter. The granular matter takes the wine-red tinge on the addition of acidulated tincture of iodine. This does not always take place with the nucleus. The cells of the hepatic patches of the amnion offer a great analogy in form and reaction to the cells of the liver when it is in a state of function.

When these cells begin to degenerate, we can see changes taking place on microscopic examination. They lose their nuclei and glycogenic matter; so that in treating a patch, which is undergoing the process of degeneration, with acidulated tincture of iodine, we have some cells giving the wine-red color and others remaining colorless; sometimes we have a medium condition, when the nucleus and granular matter has almost disappeared and the wine-red color is just perceptible.

When the patches entirely disappear they leave no trace; but when they degenerate into fatty matter, we can demonstrate the presence of fat under the microscope, at the same time that we see mixed with it beautiful octahedral crystals, presenting the characters of crystals of oxalate of lime, in the fact that they are insoluble in water and in acetic acid.

We will be struck with the constant and inverse relation which the development of the liver will bear with that of the hepatic patches of the amnios, when we compare with it the development of the liver in the embryon. During the first period of embryonic existence,* when the amniotic patches are full of glycogenic matter, the liver of the fœtus is very soft and composed of embryonic cells of a rounded or fusiform shape, which are dissolved in an alcoholic solution of potash, and are not colored by iodine. At this time we have not the least trace of glycogenic matter in the liver. When the amniotic patches have attained their full size and begin to degenerate, the glycogenic properties of the liver begin to show themselves; and that organ takes on its perfect character and functions when these patches have entirely disappeared.

This comprises the substance of Bernard's article in the *Journal de la Physiologie.* We had intended to have made our analysis much more brief; but some of the details were so interesting that we have omitted but few of them, and made nearly a full translation. The author has made similar experiments in the chick before the development of the liver, and has established the existence of glycogenic cells in the walls of the vitelline sac. The evolution of these bodies he has not yet completely investigated; and confines himself in this article to the mammalia. We will watch the progress of his experiments on this subject, and analyze any future articles which he may communicate.

*En résumé*, the following are the conclusions drawn from the preceding observations:

"1st. There exists in the placenta of the mammalia a function, which has been unknown up to the present time, and which is coincident with the absence of the glycogenic functions of the liver during the first periods of embryonic life. This temporary function is located in a transitory glandular or epithelial element of the placenta, which, in certain animals, is found mingled with the vascular portion of that organ, and which, in the ruminant animals, presents itself separately, in a way to form upon the amnios patches of an epithelial appearance, which every one is doubtless able to see, but of the glycogenic function of which we have been ignorant."

"2d. This temporary hepatic organ of the placenta, in permitting the direct study, in an isolated anatomical element, of the glycogenic matter, confirms and completes by an example what I have long said, that the formation of the glycogenic amylaceous matter is a property common to the animal and vegetable kingdoms. The observations contained in this article

* From the first periods of embryonic life, in the embryons of the calf two or three centimeters in length, I was not yet able to see the amniotic patches. Perhaps one would find glycogenic cells in the umbilical vesicle.

furnish us new analogies, inasmuch as we see the glycogenic amylaceous matter accumulate about the animal embryon and in its tissues, the same as in plants it accumulates in the grains around the vegetable embryon."

"3d. The glycogenic function in animals commences, then, from the beginning of fœtal life, and before the organ in which this function is localized in the adult is developed."

"4th. All that has been said in this article bears especially upon the glycogenic function of the liver; but actually it leads us to examine whether the biliary function which the liver possesses in the adult is equally accomplished by the hepatitic placental organ which we have described. The question should be put in these terms, to know whether the same glandular cells, are charged with the two functions, which from that time are united and connected, or if, on the contrary, the liver should not rather be considered as a complex organ, in which a combination of anatomical elements is found, which are distinct, and destined, the one for the formation of amylaceous matter and the other for the formation of bile. This question, which up to this day has not been able to be resolved by anatomists, in spite of the numerous works of which the liver has been the subject, seems to me able to be elucidated and even decided by physiological researches, made, on the one part, upon the embryonic development of the function, and on the other part, on the inferior animals. I have undertaken some experiments on this subject, of which I will give an account to the *Académie* as soon as they are finished."

